# Should Coffee Consumers Not Have Their Habitual Morning Coffee Before Testing Their Cognitive Functions?

**DOI:** 10.1002/hup.70070

**Published:** 2026-10-24

**Authors:** Nikolaos Pitsikas, Duc Truong Nguyen, Arjan Blokland

**Affiliations:** ^1^ Department of Neuropsychology and Psychopharmacology Faculty of Psychology and Neurosciences Maastricht University Maastricht the Netherlands; ^2^ Division of Physical Activity, Cancer Prevention and Survivorship German Cancer Research Center (DKFZ) and University Medical Center Heidelberg Heidelberg Germany

**Keywords:** caffeine, reaction time, Stroop task, verbal memory, working memory

## Abstract

**Objective:**

Caffeine could improve cognitive functions, but study protocols usually prohibit drinking coffee before cognitive testing. However, regular coffee consumers may experience withdrawal effects when they cannot drink their habitual coffee, which may impair cognitive performance. Therefore, we tested the effects of overnight coffee abstinence in regular coffee consumers: once allowing to drink their coffee and once not drinking coffee. The cognitive performance was compared with non‐regular coffee consumers.

**Methods:**

Thirty regular coffee consumers (RCCs) and 25 non‐regular consumers (N‐RCCs) took part in this study. RCCs were tested after their habitual morning coffee (c‐RCC) and after overnight abstinence (a‐RCC); N‐RCCs were tested once. A cognitive battery assessed reaction time (RT), processing speed (digit symbol substitution test), working memory, verbal learning/memory and executive function (Stroop). Sleep quality was included as a covariate.

**Results:**

Compared to c‐RCC, overnight coffee abstinence significantly slowed RTs but did not affect the performance in the other cognitive tasks. In the a‐RCC condition, but not the c‐RCC condition, the RTs were slower than those of the N‐RCCs, while accuracy measures were comparable across groups. Sleep quality did not alter these findings.

**Conclusions:**

Overnight coffee abstinence selectively slowed RT, and no statistical support was found for effects on memory or executive function. This suggests that overnight coffee abstinence protocols may introduce withdrawal‐related RT slowing as a confound in habitual consumers, whereas other cognitive domains may not be affected. Allowing habitual consumers to maintain normal coffee intake could reduce this bias, supporting tailored rather than uniform abstinence requirements based on the specific outcomes studied.

## 1 Introduction

Coffee is one of the most widely consumed beverages in the world. Caffeine is the major psychoactive component of coffee and is well‐known for its mild stimulatory properties (Higdon and Frei 2006). Therefore, coffee's popularity is not simply driven by the taste, but also by the stimulatory action of it (Ágoston et al. 2018). A substantial body of research suggests that caffeine—the main ingredient of coffee—may exert beneficial effects on several cognitive domains, including attention, memory and executive functioning (Caldenhove et al. 2018; Jarvis 1993; Magalhaes et al. 2021; Nehlig 2010; Ricupero and Ritter 2024).

Specifically, caffeine has been shown to improve performance on reaction‐time tasks, likely through enhanced vigilance and attentional processing (Fisone et al. 2004; Ricupero and Ritter 2024). Attention is a fundamental cognitive function that supports a range of higher‐order processes, including working memory, inhibitory control and cognitive flexibility. Consequently, improvements in attention may indirectly influence performance across multiple cognitive domains. Evidence further suggests that caffeine's cognitive effects are dose‐dependent, with moderate doses (100–300 mg) generally associated with improved vigilance and reaction time performance (Raise‐Abdullahi et al. 2024; Ricupero and Ritter 2024), whereas higher doses (> 400 mg) may produce adverse effects (e.g., Liu et al. 2024; Nehlig 2010).

The stimulatory effects of caffeine are primarily attributed to its antagonistic action at adenosine receptors (Fisone et al. 2004). Adenosine normally exerts inhibitory effects on neuronal activity by reducing the release of several neurotransmitters, including glutamate, acetylcholine, serotonin, noradrenaline and dopamine (McLellan et al. 2016). As a competitive antagonist at both A_1_ and A_2A_ adenosine receptors, caffeine counteracts these inhibitory actions, thereby facilitating neurotransmitter release and increasing neuronal activity. These neurochemical effects are thought to underlie the observed enhancements in alertness, attention and psychomotor vigilance following caffeine consumption (Huang et al. 2005; McLellan et al. 2016).

In clinical and experimental research, cognitive assessments are typically conducted under conditions designed to minimise the influence of external factors that may confound performance. Participants are therefore often instructed to abstain from substances such as alcohol, nicotine and caffeine prior to testing. While this approach aims to ensure standardised baseline measurements, it may introduce a methodological challenge in studies involving habitual caffeine consumers (James 2014).

Coffee abstinence can induce a withdrawal syndrome characterised by symptoms such as headache, fatigue, decreased subjective energy and well‐being, irritability and difficulty concentrating (Juliano et al. 2012). These symptoms generally emerge within 12 hours of caffeine cessation and vary in severity according to habitual caffeine intake (Juliano and Griffiths 2004). As several withdrawal symptoms directly affect cognitive and attentional functioning, cognitive performance measured during periods of caffeine abstinence may not accurately reflect an individual's typical level of functioning.

Indeed, it has been proposed that the vigilance and psychomotor benefits attributed to caffeine in regular coffee consumers may largely reflect the reversal of withdrawal symptoms rather than genuine effects on these functions beyond baseline levels (James and Rogers 2005; Rogers et al. 2005, 2013). According to this hypothesis, caffeine restores performance in regular coffee consumers (RCCs) to levels comparable with those observed in non‐regular consumers (N‐RCCs), rather than producing superior performance. Consequently, when RCCs are required to abstain from coffee before cognitive testing, their performance may be negatively affected due to withdrawal‐related effects, potentially confounding the interpretation of cognitive outcomes.

This issue may be particularly relevant among RCCs who report that they require their morning coffee to function optimally. For such individuals, cognitive performance assessed after overnight caffeine abstinence may differ substantially from performance observed after consuming their habitual morning coffee. Examining these conditions within the same individuals may therefore provide insight into the extent to which overnight coffee abstinence influences cognitive functioning. Furthermore, comparing the performance of RCCs under abstinence and non‐abstinence conditions with that of N‐RCCs may help clarify whether any observed deficits reflect abstinence‐induced impairment rather than enduring differences in cognitive ability.

The present study was designed to address these questions. Specifically, we investigated the effects of overnight coffee abstinence in RCCs who self‐reported needing their morning coffee to achieve optimal cognitive performance. Participants completed a battery of behavioural tasks assessing memory, reaction time, attention and executive functions under conditions of overnight coffee abstinence and habitual coffee consumption. In addition, the performance of the RCCs (with or without their habitual morning coffee) was compared with that of N‐RCCs to determine whether acute coffee abstinence results in measurable cognitive impairments relative N‐RCCs.

## 2 Methods

### 2.1 Participants

We included two participant groups: non‐regular coffee consumers (N‐RCC; *n* = 25) and regular coffee consumers (RCC; *n* = 30). A power analysis showed that for testing the within‐subject effects (abstinence‐RCC vs. coffee‐RCC)—on the basis of a medium effect size (*d*_*z*_ = 0.50) and a power of 0.80, 28 participants were required. This was based on the reaction time task with 3 levels of difficulty (see below). For the between‐subject analysis (N‐RCC vs. the a‐RCC and the c‐RCC condition), the power calculation revealed the same required sample size. Therefore, the total sample size in this study was considered sufficient. The participant recruitment took place via advertisements. Exclusion criteria for both groups were severe visual impairments (glasses and contact lenses were permitted) and colour blindness. The age inclusion criterion ranged from 18 to 29 years. Inclusion criteria for the RCC group consisted of drinking at least two cups of coffee per day, of which at least one cup of coffee is consumed in the morning. Consuming two cups of coffee per day, each containing an average of 80–100 mg of caffeine, aligns with the criteria for habitual caffeine intake (Addicott et al. 2009). The inclusion criteria for the N‐RCC group were defined by consuming a maximum of one cup of coffee per day and not regularly consuming coffee in the morning on a regular basis. The study was approved by the local Ethical Committee of the Faculty of Psychology and Neuroscience of Maastricht University.

### 2.2 Procedure

RCC participants were recruited with an advertisement text saying: ‘Do you need coffee in the morning?’ Participants who signed up for this study first completed a pre‐screening questionnaire. The main goal of this study was to provide insight into the actual coffee consumption and timing of coffee consumption. N‐RCCs were recruited with an advertisement saying: ‘We need participants who do not drink coffee in the morning’. Participants (Psychology students) received study credits for participating in the study.

The RCC group was tested twice. They were tested when they had their regular coffee in the morning (c‐RCC condition) and after overnight coffee abstinence (a‐RCC condition). In the case of the a‐RCC condition, participants were instructed to refrain from consuming coffee after 18:00 on the day preceding the testing session. The order of the a‐RCC and the c‐RCC conditions were randomised for the RCC participants. Both test days were separated by a week. The N‐RCC group was only tested once. For both groups, testing took place in the morning between 8:30 and 11:00. For the RCC participants, the timing was dependent on their usual morning coffee consumption timing. They were tested 1–2 hours after their usual coffee consumption.

On the day of testing, the participants gave their informed consent to participate in the study. Subsequently, participants completed the Groninger Sleep Quality Scale (GSQS) to assess their sleep quality (Jafarian et al. 2008). Scores can range between 0 and 14, in which a high score indicates poor sleep quality. Participants were also asked about the number of cups of coffee consumed in the morning. Finally, the participants were asked whether they experienced coffee cravings before they were tested. The latter question offered responses on a 5‐point scale ranging from ‘not at all’ (1) to ‘very much’ (5).

Tests were carried out using a computer and took place in a controlled environment, in which a researcher was present to give instructions and was available for queries when needed.

### 2.3 Cognitive Test Battery

The computerised cognitive test battery consisted of a reaction time (RT) task, the Digit Symbol Substitution Test (DSST), the n‐Back Task, the Visual Verbal Learning Test (VVLT) and the Stroop Colour andWord Test (Stroop).

### 2.4 Reaction Time Test

In the RT task (Caldenhove et al. 2018), participants were presented with a response box consisting of a row of five square buttons, each with a corresponding round light positioned directly above it. The task consists of three parts. In the first part (simple RT), participants had to keep pressing down the middle button until they heard a signal sound. Then, once the light over the middle button lit up, participants had to release their fingers from the button as quickly as possible. The time between lighting up and finger release was measured as RT. Then the choice RT followed. Here, participants were instructed to keep pressing down the middle button until they heard an auditory signal. This time, however, one of the outer lights (i.e., either left or right) would light up. In response, participants had to lift their fingers from the middle button and press the button corresponding to the lit‐up light as fast as possible. Finally, in the last part (incompatible RT), participants had to press the button on the opposite side of the light (i.e., the outer left light and the outer right button). For the choice and incompatible RT, the time between lighting up and pressing the corresponding button was measured as RT.

### 2.5 Digit Symbol Substitution Test

At the top of a computer screen, a line of nine boxes was displayed, each filled with a different symbol and each symbol was associated with a number from 1 to 9 (Jaeger 2018). In the middle of the screen, numbers were displayed sequentially and one by one. At the bottom of the screen, a line of randomised symbols was displayed. For each of the numbers displayed in the middle, the participant had to match the corresponding symbol at the bottom by clicking on them. The number of correct responses made within 3 min was recorded.

### 2.6 n‐Back Task

In this test, a serial sequence of letters is presented to the participant who should indicate whether the letter shown is the same as the letter displayed *n* trials earlier (Jaeggi et al. 2010). The participant indicated whether the shown letter is the same or different as the letter presented *n* trials ago by pressing the ‘/’ key for the same letter and the ‘z’ key if a different letter is shown. The participants completed two versions: a 1‐Back and 3‐Back task. For both versions, 10 practice trials preceded the actual task to ensure a complete comprehension of the instructions. An interval of 5 min between the two versions of the n‐Back test was given. The response accuracy (i.e., percentage correct) and the reaction time were recorded.

### 2.7 Visual Verbal Learning Test

The participant was presented with a sequence of 30 monosyllabic English words and was prompted to remember as many as possible (Brand and Jolles 1985). On the computer screen, each word was automatically presented one by one. Each word was presented for 1 s followed by a 1 s interval. After the list of 30 words were presented, the participant was prompted to vocally recall as many words as possible. This procedure was repeated three times. About 20 min later, during which the participants were doing other tests, the participant was again asked to recall as many words as possible, but without being given another presentation of the words. The number of the correctly recalled words after each presentation (immediate recall), as well as the number of the correctly recalled words 20 min after their presentation (delayed recall) was recorded.

### 2.8 Stroop Task

This task is divided into three parts, with each part allotted a duration of 45 s (Stroop 1935). In principle, a participant had to read a sheet containing a word list of 50 English colour names. In case a participant finished reading the page within the 45 s, they were instructed to continue from the beginning of the page. This instruction was given before each part. In this first part, the colour words were written in a black font colour. In the second part, instead of colour names, 50 coloured rectangles were shown, and participants had to name the colour of these rectangles. In the last part, colour words were presented again, but each word being written in different font colour. Instead of reading out the word, participants had to name the font colour and not the word itself. Colours used in this procedure were red, blue, green and yellow. For all parts, participants are instructed to read as fast as possible and avoid making mistakes. The number of correct responses within 45 s time limit was recorded. In addition, the interference score was calculated by subtracting the predicted colour‐word from the observed number of colour‐words (see Golden 1978).

### 2.9 Statistical Analysis

The analyses were conducted using JASP. For every parameter, the mean and standard error of the mean was calculated for both groups and RCC conditions. Exceptions were made for RT data, where RT task medians per participant were calculated first, and then were used to calculate group means. This was done because the RT data were not normally distributed. First, the effects of coffee abstinence were evaluated in the RCC group. DSST and the delayed VVLT were analysed by using paired sample *t*‐tests. All other cognitive tests, namely n‐Back, immediate VVLT, RT and Stroop, were analysed by using repeated measures GLM with the within‐subject factors Coffee condition and the respective Test trials. Additionally, craving scores, cups of coffee and GSQS scores on testing days were analysed using a paired samples *t*‐test. Second, the performance of each RCC conditions was compared with the N‐RCC group. The same approach as mentioned above was used. Finally, analyses were run with sleep quality as a covariate in order to check if sleep affected the performance in the different tasks. In case there was only one data point in the N‐RCC group, Pearson correlations were used to examine the relation between the performance and sleep quality.

For all main and interaction effects, an *α* level of 0.05 was applied. In the case of post‐hoc analyses with multiple comparisons, a Šidák correction was used when comparing the a‐RCC and c‐RCC conditions, and a Bonferroni when comparing between N‐RCC and RCC conditions.

## 3 Results

The participants' age, coffee use and sleep quality data are shown in Table 1. As expected, N‐RCC participants did not manifest any sign of craving as their score on the craving question was 0. However, one N‐RCC scored 4 on the craving scale and was deleted from further analysis. In addition, the N‐RCC group as well as the participants in the a‐RCC condition drank no coffee in the morning of the test day, whereas the participants in the c‐RCC condition drank almost 2 cups of coffee on average. The RCC participants showed higher craving scores before the a‐RCC condition as compared to c‐RCC condition. Sleep quality for the night preceding the test sessions revealed no differences among the various groups and conditions.

**TABLE 1:** Participant characteristics for the different groups.

	N‐RCC (*n =* 24)	a‐RCC c‐RCC (*n =* 30)	
Age (years)	22.9 ± 0.5	22.8 ± 0.4	—
#Morning coffee cups	0 ± 0	0 ± 0	1.9 ± 0.13*^,^**
Craving (score)	1 ± 0	3.2 ± 0.18*	1.6 ± 0.10**
GSQS (score)	3.8 ± 0.59	4.2 ± 0.57	4.4 ± 0.70

*Note:* Data were expressed as means ± stderr.

Abbreviations: a‐RCC, acute abstained regular coffee consumers; c‐RCC, regular coffee consumers; GSQS, Groninger Sleeping Questioannire Scale; N‐RCC, non‐regular coffee consumers.

^*^ *p* < 0.01: different from N‐RCC.

^**^ *p* < 0.01 different from a‐RCC.

### 3.1 Effects of Coffee Abstinence: Comparing c‐RCC and a‐RCC

Table 2 shows the performance of the different groups and conditions in a battery of cognitive tests.

**TABLE 2:** Overview of the performance of the N‐RCC and RCC groups and conditions on the different cognitive tasks.

Cognitive test	N‐RCC	a‐RCC	c‐RCC
	(*n =* 24)	(*n =* 30)	
Simple RT test (ms)	122.8 ± 10.3	116.2 ± 6.7	103.1 ± 6.4
Choice RT test (ms)	134.2 ± 7.3	194.6 ± 14.5	167.7 ± 12.5
Incompatible RT test (ms)	161.7 ± 8.3	208.3 ± 17.9	181 ± 12.8
DSST (score)	88.2 ± 2.1	88.4 ± 2	88.6 ± 2.1
1‐Back accuracy (%correct)	96.7 ± 0.5	96.3 ± 0.6	95.8.1 ± 0.7
3‐Back accuracy (%correct)	85.4 ± 1.8	82.7 ± 1.7	82.8 ± 1.8
1‐Back RT (ms)	576.5 ± 20.5	561.2 ± 13.8	543.5 ± 16.6
3‐Back‐RT (ms)	850.1 ± 37.7	832.9 ± 34.8	875.3 ± 36.8
VVLT1 (number of words)	12.2 ± 0.7	11.8 ± 0.7	12.6 ± 0.7
VVLT2 (number of words)	18.5 ± 0.9	17.4 ± 0.9	18.2 ± 0.7
VVLT3 (number of words)	21.6 ± 1	21.1 ± 0.9	22 ± 0.8
VVLT delayed (number of words)	20.1 ± 1.2	19.4 ± 0.9	19.8 ± 0.7
Stroop reading test (number of words)	115.2 ± 3.3	108.3 ± 3.2	110.1 ± 4
Stroop colour test (number of words.)	83.4 ± 2.8	81.1 ± 2.4	85.9 ± 2.8
Stroop invalid test colour (number of words)	60.2 ± 2.0	63.1 ± 1.8	64.5 ± 2.0
Stroop interference score	16.0 ± 2.9	17.1 ± 1.8	16.7 ± 1.4

*Note:* Data were expressed as means ± standard errors.

Abbreviations: a‐RCC, acute abstained regular coffee consumers; c‐RCC, regular coffee consumers; DSST, Digit Symbol Substitution Test; *n*, number of participants; N‐RCC, non‐regular coffee consumers; RT, reaction time; VVLT, Visual Verbal Learning Test.

### 3.2 Reaction Time Test

There was no interaction between coffee abstinence and RT performance [*F*(2, 58) = 2.10, n.s., *η* ^2^ = 0.14; see Figure 1A]. However, significant main effects of the RT conditions [*F*(2, 58) = 43.66, *p* < 0.001, *η* ^2^ = 0.62], and coffee abstinence [*F*(1, 29) = 17.54, *p* < 0.001, *η* ^2^ = 0.38] were evident. Post‐hoc analyses showed that the simple RT were faster than the choice and incompatible reaction times (both *p* < 0.001), and that the incompatible RT was slower than the choice RT (*p* < 0.05).

**FIGURE 1:**
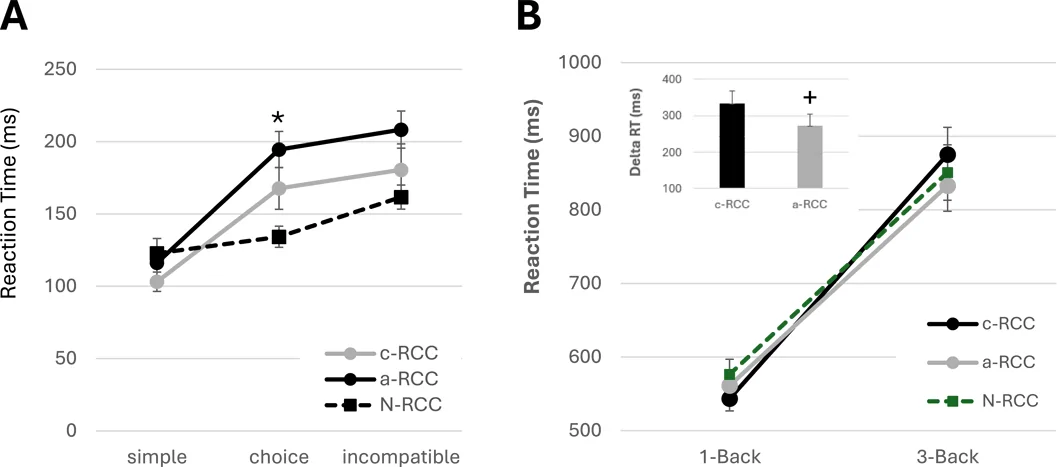
Performance of the RCC and N‐RCC on a reaction time task (A) and the RT in the n‐back task (B). The insert in the right panel represents the difference score of RT (3‐back–1‐back). Data represent mean and stderr. *: *p* < 0.01 (N‐RCC vs. a‐RCC); +: *p* < 0.05.

### 3.3 Digit Symbol Substitution Test

There was no difference between the c‐RCC and the a‐RCC condition in the number of symbols that were correctly scored (t(29) = 0.13, n.s., Cohen's *d* = 0.024).

### 3.4 n‐Back‐Task

The percentage correct trials on the 1‐Back task was higher when compared to the 3‐Back task [*F*(1, 29) = 67.19, *p* < 0.001, *η* ^2^ = 0.70]. There was no effect of the coffee abstinence [*F*(1, 29) = 0.08, n.s. *η* ^2^ = 0.003)], nor a significant interaction between n‐Back trials and coffee abstinence condition [*F*(1, 29) = 0.69, n.s., *η* ^2^ = 0.006].

The reaction times in the 3‐back task were slower when compared to the 1‐back task [*F*(1, 29) = 87.27, *p* < 0.001, *η* ^2^ = 0.75; see Figure 1B]. There was an interaction between the load of the n‐Back task and coffee abstinence condition (*F*(1,29) = 6.15, *p* < 0.05, *η* ^2^ = 0.18), indicating that the increase in RT from the 1‐Back to 3‐Back was greater in the c‐RCC condition as compared to the a‐RCC condition (see inserted panel Figure 1B). There was no difference between the overall RT of the c‐RCC and a‐RCC condition [*F*(1, 29) = 0.57, n.s., *η* ^2^ = 0.01].

### 3.5 Visual Verbal Learning Test

The number of words recalled increased during the three immediate recall trials [*F*(2, 58) = 233.85, *p* < 0.001, *η* ^2^ = 0.89]. There was no difference between the c‐RCC and a‐RCC conditions [*F*(1, 29) = 2.51, n.s., *η* ^2^ = 0.08], nor an interaction with Trials [*F*(2, 58) = 0.97, n.s., *η* ^2^ = 0.001].

Also, for the delayed recall performance, no differences were observed between c‐RCC and a‐RCC condition [t(29) = 0.42, n.s., Cohen's *d* = 0.08].

### 3.6 Stroop Test

With increasing task difficulty, fewer words were read from the list [*F*(2, 58) = 112.69, *p* < 0.001, *η* ^2^ = 0.80]. There was no effect of coffee abstinence [*i*(1, 29) = 3.00, n.s. *η* ^2^ = 0.09], nor an interaction effect with task difficulty [*F*(2, 25) = 1.72, n.s., *η* ^2^ = 0.06]. The interference score did not differ between the two conditions (*F*(1, 29) = 0.10, n.s. *η* ^2^ = 0.003).

### 3.7 Comparing N‐RCC versus c‐RCC and a‐RCC

#### 3.7.1 Reaction Time Test

There were two occasions where the test did not run properly during the RT task, and data of two participants from the N‐RCC group were excluded from the analysis. When comparing the N‐RCC group with the c‐RCC condition, a clear interaction was found with increasing task difficulty [*F*(2, 100) = 10.04, *p* < 0.001, *η* ^2^ = 0.17; see Figure 1A]. For the simple RT, the c‐RCC reacted faster, but the participants in the c‐RCC condition were slower on the choice and incompatible RT task. In a similar way, this interaction was found when the a‐RCC condition was compared with the N‐RCC group [*F*(2, 100) = 11.41, *p* < 0.001, *η* ^2^ = 0.19]. No main effect of group was found when comparing the c‐RCC condition with the N‐RCC group [*F*(1, 50) = 0.41, n.s., *η* ^2^ = 0.01]. However, an overall slower RT was found for the a‐RCC condition when compared with the N‐RCC group [*F*(1, 50) = 4.56, *p* < 0.05, *η* ^2^ = 0.08]. When comparing the RT performance for each condition, only a difference between the N‐RCC and the a‐RCC was found for the choice reaction time task [*t*(50) = 3.34, *p* < 0.01, Cohen's *d* = 0.94].

#### 3.7.2 Digit Symbol Substitution Test

A *t*‐test revealed no difference between the N‐RCC and the c‐RCC condition [both *t* (52) < 0.90, n.s., Cohen's *d* < 0.04].

#### 3.7.3 n‐Back‐task

There were two occasions where the test did not run properly during the 1‐Back task, and data of two participants from the N‐RCC group were lost. When comparing the N‐RCC group with the c‐RCC condition, no interaction in the response accuracy was found [*F*(1, 50) = 0.58, n.s., *η* ^2^ = 0.004]. This was also found when comparing the N‐RCC group with the a‐RCC condition [*F*(1, 50) = 0.58, n.s., *η* ^2^ = 0.002]. In addition, no main effect of coffee was found when comparing the N‐RCC group with the two RCC conditions [both *F*(1, 50) < 0.08, n.s., *η* ^2^ < 0.002].

For the RT, there was no interaction between the N‐RCC group and the c‐RCC or the a‐RCC condition and n‐Back level [both *F*(1, 50) < 0.31, n.s., *η* ^2^ < 0.02], nor a main effect of coffee for both comparisons [both *F*(1, 50) < 0.32, n.s., *η* ^2^ < 0.001].

#### 3.7.4 Visual Verbal Learning Test

When comparing the N‐RCC group with the c‐RCC and the a‐RCC condition, no interaction between trials and groups was found [both *F*(2, 104) = 0.59, n.s. *η* ^2^ = 0.01]. In addition, no main effect of group was observed for both comparisons [both *F*(1, 52) < 0.34, n.s., *η* ^2^ = 0.01].

Regarding the VVLT delayed recall, the number of words recalled in both comparisons of the N‐RCC and RCC conditions was similar [both *t*(52) < 0.43. n.s., Cohen's *d* = 0.12].

#### 3.7.5 Stroop Test

For the interference score, two observations in the NRCC group were identified as outliers (> 2.5 standard deviations) and were therefore not included in the analysis. Comparison of the performance of the RCC group with the c‐RCC and the a‐RCC condition in this task did not show an interaction between tasks and group [*F*(2, 104) = 2.43, n.s. *η* ^2^ = 0.05]. In addition, no main effect of group was observed when comparing the N‐RCC group with the RCC conditions [*F*(1, 52) = 0.58, n.s., *η* ^2^ = 0.01]. For the interference score, no difference between the NRCC and the RCC conditions was observed (*F*(2, 79) = 0.07, n.s., *η* ^2^ = 0.02).

#### 3.7.6 Sleep Quality as Covariate

Overall, the covariate analysis did not affect the main outcomes on the effects found without the covariate.

When using sleep quality as a covariate in the different analyses for the RCC group, it was found that sleep did not interact with the RT task (all *F*'s < 3.44), n‐Back accuracy (all *F*'*s* < 1.08), DSST (*F*'*s* < 0.55), VVLT immediate (*F*'*s* < 2.26) and delayed recall (all *F*'s < 0.10). However, sleep quality interacted with the RT task difficulty in the n‐Back task (*F*(1, 28) = 4.97, *p* < 0.05, *η* ^2^ = 0.15) and had a main effect on the RT in the n‐Back task (1,28) = 9.31, *p* < 0.01, *η* ^2^ = 0.25). Examining the interaction between n‐Back level and sleep, it was found that there was a negative correlation between sleep quality and RT in the 3‐Back task (c‐RCC condition: *r*_*p*_ = 0–51, *p* < 0.01; a‐RCC condition: *r*_*p*_ = −0.36, *p* = 0.05). The negative correlations indicated that poor sleep was associated with faster reaction times. There were no correlations between RT and sleep quality in the 1‐Back task (*r*_*p*_'s < −0.23). Finally, there was a main effect of sleep on the performance in the Stroop test (*F*(1, 28) = 7.93, *p* < 0.01, *η* ^2^ = 0.22). A closer inspection of the data showed overall positive correlations between sleep and Stroop performance (0.23 < *r*_*p*_ < 0.32), where the colour reading condition in the c‐RCC condition strongly correlated with the sleep quality (*r*_*p*_ = 0.48, *p* < 0.01). These data indicate that the overall lower sleep quality was associated with more words being read.

In the N‐RCC group, no interactions or overall effects of sleep quality were found (all *F*'*s* < 3.12). In addition, no correlations between sleep quality and DSST and delayed recall were found (*r*_*p*_'*s* < 0.27).

## 4 Discussion

Our first aim was to determine whether overnight coffee abstinence in RCCs affects cognitive performance. The results indicate that there was no statistical support that overnight coffee abstinence did impair performance across the cognitive domains assessed. However, RTs the RT test were generally slower when RCC participants after overnight coffee abstinence. Furthermore, compared with the N‐RCC group, participants in the a‐RCC condition exhibited slower RTs, suggesting that overnight coffee abstinence primarily affected psychomotor functions rather than general cognitive functioning.

Previous studies have shown that coffee abstinence resulted in lower levels of vigilance and psychomotor performance (Evans and Griffiths 1999; Ratliff‐Crain et al. 1989; Rogers and Dernoncourt 1998; Rogers et al. 2013; Rogers et al. 1995). Our finding that coffee abstinence slowed down reaction time responding is in line with these earlier studies. In the current study, the coffee abstinence was about 14 h, which would fall in the time window where withdrawal symptoms could be observed, although the peak intensity is found after 20–51 h (Juliano and Griffiths 2004). Although no physical and mood withdrawal symptoms were assessed in the current study, the subjective craving was clearly higher in the a‐RCC condition than the c‐RCC condition. This indicates that the a‐RCC was sufficient to induce coffee craving in the RCC participants. Although longer abstinence periods could have stronger effects (Rogers et al. 2005, 2013), the aim of the current study was to assess whether not allowing participants drinking their morning coffee affected their cognitive performance when tested in the morning.

Although we found slower reaction times when RCC participants abstained from coffee, we found no statistical evidence of a difference in response accuracy between the a‐RCC and c‐RCC conditions or in comparisons with the N‐RCC group. The reaction‐time finding nevertheless indicates that an aspect of task performance differed between conditions. Therefore, the absence of a detected accuracy difference should not be interpreted as evidence that cognitive performance was unaffected. We did not formally assess the relationship between response speed and accuracy, so we cannot determine whether changes in response strategy contributed to the reaction‐time findings. In particular, the slower responses were not accompanied by a statistically detectable improvement in accuracy, but this does not rule out a speed–accuracy trade‐off.

To the best of our knowledge, the effects of overnight coffee abstinence on memory and executive functions have not been thoroughly investigated in experimental studies. Some studies investigated the effects of coffee withdrawal on memory and found effects on meta‐memory (Palmer et al. 2017) and working memory (Lin et al. 2023). However, the withdrawal effects were not investigated in participants who were regular coffee drinkers but were related to conditioning to caffeine intake. One study that also included regular coffee drinkers who had to abstain from drinking their regular coffee did not find any effect on working memory (Sevenoaks et al. 2026). It should be mentioned that our design was focused on the effects of overnight coffee abstinence on cognitive performance in the morning since this is a common practice in our studies. It can be anticipated that if participants have to abstain from drinking coffee longer, when tested later on the day, the withdrawal effect would be at least similar. In addition, it cannot be excluded that coffee withdrawal does affect cognitive performance when tested after a longer withdrawal period (20–51 h) (see Juliano and Griffiths 2004).

Our second aim was to determine whether cognitive performance differed between the RCC conditions and the N‐RCC group. The RT test results demonstrated that both RCC conditions differed from the N‐RCC group. Specifically, participants in both RCC conditions responded faster during the simple reaction time task but slower during the choice and incompatible reaction time tasks. Notably, the difference between the N‐RCC and a‐RCC groups was particularly pronounced for the choice reaction time task. These findings suggest that psychomotor performance remains altered in both RCC conditions compared with the N‐RCC group. In contrast, RT performance on the n‐back task was not affected, indicating that the differences between the RCC and N‐RCC groups were task‐specific rather than reflecting a generalised slowing or speeding of responses.

Because coffee consumption can influence both sleep duration and sleep quality (Chaudhary et al. 2016; Clark and Landolt 2017), and sleep quality is closely associated with cognitive functioning (Kronholm et al. 2009; Wild et al. 2018), we also examined the relationship between sleep quality and cognitive performance in both groups. Sleep quality was not associated with cognitive performance in the N‐RCC group. However, significant interactions were observed for RT in the n‐Back task and performance in the Stroop tasks.

Contrary to previous studies reporting that poorer sleep quality and shorter sleep duration are generally associated with slower RTs and reduced Stroop task performance (Takeuchi et al. 2018; Wild et al. 2018), our findings suggest that poorer sleep quality, in combination with habitual coffee consumption, was associated with improved performance on these tasks (but see Zavecz et al. 2020). These findings should be interpreted with caution, however, as the mean Groningen Sleep Quality Scale (GSQS) score in our sample was relatively low (approximately 4). Given that GSQS scores above 6 are typically considered indicative of poor sleep quality, the limited variability and generally good sleep quality in our sample may have reduced the reliability of these associations or contributed to the unexpected direction of the observed effects. On the other hand, the finding that sleep seemed to interact with the performance in the RCC conditions but not in the N‐RCC group may suggest that sleep quality and habitual coffee intake seem to have additive effects that affect some cognitive functions.

In clinical and experimental research, cognitive assessments are typically conducted under standardised conditions to minimise the influence of external factors that may confound performance. Accordingly, participants are often instructed to abstain from substances such as alcohol, nicotine and caffeine before testing. Although this approach is intended to establish a standardised baseline, it may inadvertently introduce bias in studies involving RCCs (James 2014). The present findings suggest that overall, the performance of the RCCs was more similar to that of N‐RCCs when they had consumed their usual morning coffee (c‐RCC condition). When the RCCs were subjected to overnight coffee abstinence (a‐RCC condition), the accuracy performance was not affected but slowed the RT performance in the RT and n‐Back task. Consequently, protocols that require to abstain from drinking coffee in the morning, which intends to control for the effects of caffeine, may actually have an adverse effect when testing RT performance in RCCs.

Although some differences were found between the RCC and NRCC, these differences cannot only be related to differences in drinking the morning coffee. First, the effects of overnight coffee abstinence was tested in a within‐subjects design where participants were tested twice, whereas the NRCC group was tested only once. Therefore, other factors may explain the differences between the groups (e.g., training effect). In addition, several studies have shown that habitual coffee consumption leads to differences in the gut and brain (Boscaini et al. 2026; Magalhaes et al. 2021), which could be explained by changes in the microbiome and the brain (Boscaini et al. 2026; Lopes et al. 2023). Therefore, the comparison between the RCC and NRCC should not be interpreted in terms of acute coffee consumption.

Several limitations could be considered when interpreting the current findings. First, our sample was restricted to university students, which limits the generalisability of our results to other populations. Second, coffee intake was assessed via self‐report, which may not accurately capture actual consumption given variability in cup size, brewing method and caffeine content. Third, we did not biochemically verify abstinence (e.g., via plasma or saliva caffeine levels), so we cannot confirm participant compliance or rule out the influence of residual caffeine at the time of testing. A fourth limitation could be that we did not screen the participants for consuming other products containing caffeine (e.g., tea or energy drinks). This could have caused unexpected differences in caffeine levels between the conditions and groups. Finally, we did not directly assess withdrawal symptoms, relying instead on self‐reported cravings as a proxy; consequently, we cannot determine whether participants actually experienced withdrawal or whether cognitive performance was related to withdrawal severity in a dose‐dependent manner. Future studies incorporating biochemical verification and validated withdrawal measures are needed to clarify these relationships.

## 5 Conclusions

In summary, the present study found no statistical support that overnight coffee abstinence in RCCs substantially impairs cognitive performance across the domains assessed. However, overnight coffee abstinence was associated with slower RTs. These findings have two methodological implications. First, caffeine abstinence may confound RT performance in studies including RCCs. Second, allowing RCCs to consume their usual morning coffee before testing may minimise withdrawal‐related effects on psychomotor performance without affecting the accuracy performance on the cognitive domains tested in this study. Accordingly, pre‐test caffeine abstinence protocols should be tailored to the specific cognitive outcomes of interest, rather than applied uniformly across studies.

## Author Contributions

A.B. designed the study. D.T.N., and N.P. analysed the data. N.P., D.T.N., and A.B. wrote the manuscript.

## Funding

The authors have nothing to report.

## Ethics Statement

The study was approved by the Ethical Research Committee of the Faculty of Psychology and Neuroscience (ERCPN‐263_21_02_2023), and all participants gave consent to participate in the study.

## Conflicts of Interest

The authors declare no conflicts of interest.

## Data Availability

The data can be obtained upon request.
